# Nucleus management with Blumenthal technique: Anterior chamber maintainer

**DOI:** 10.4103/0301-4738.58487

**Published:** 2010

**Authors:** Rupesh Agrawal

**Affiliations:** Ocular trauma, Uveitis and Comprehensive Ophthalmology, L V Prasad Eye Institute Hyderabad, India

Dear Editor,

I read with interest the article by Malik *et al*. on nuclear management with Blumenthal technique[1] and in the context of the article would like to comment on a few points. The article is titled “Nucleus management with Blumenthal technique”, however, in the whole article the original technique described by the late Prof Michael Blumenthal is missing.

Blumenthal had elegantly described the use of anterior chamber maintainer in extracapsular cataract extraction and manual small-incision cataract surgery (SICS).[2–4] In the paragraph on nuclear delivery,[1] the authors have stated the use of viscoelastic to facilitate nucleus delivery[1] but the original Blumenthal technique described the use of viscoelastic agent only in difficult cases, in case of complications during manual SICS.[2–4] According to Blumenthal, anterior chamber maintainer in itself provides sufficient anterior chamber depth and fluidics in facilitating nucleus prolapsed in anterior chamber and subsequently, Sheet's glide aids in smooth atraumatic delivery of nucleus.[4]

Blumenthal has described use of smooth round-tipped Blumenthal cannula for hydrodissection which is very different from the conventional sharp-tipped hydrodissection cannula. The original hydrodissection technique described by Blumenthal entails the hydrodissection of superior 12 o'clock pole through side port incision by Blumenthal cannula followed by supplemented hydrodissection at 3 o'clock and 9 o'clock pole which facilitates superior pole of the nucleus to pop out of the bag which is subsequently pushed out into the anterior chamber followed by maneuvering out the remaining nucleus with the help of the Blumenthal canula only. At no step is the use of viscoelastic agent advocated.[3]

Similarly, during removal of the cortex Blumenthal has again described the use of single-port cannula along with anterior chamber maintainer and has not advocated use of viscoelastic agent. The diagrammatic representation showing the frown incision in Fig. 1 is also not described by Blumenthal, he has advocated the use of straight incision of maximum size 5 mm with back cuts on either side of straight incision of length up to 1.5 mm, with 100-110 degrees angulation with straight incision, and further dissection of the tunnel under anterior chamber maintainer only without using viscoelastic agent.[3]

I have been practicing and teaching the original Blumenthal technique since the last six years and have been fairly successful in using the originally described Blumenthal technique.
